# How to Take Autophagy and Endocytosis Up a Notch

**DOI:** 10.1155/2014/960803

**Published:** 2014-04-22

**Authors:** Julia M. I. Barth, Katja Köhler

**Affiliations:** ^1^Department of Biology, Institute of Molecular Systems Biology, ETH Zürich, 8093 Zürich, Switzerland; ^2^Department of Biosciences, Centre for Ecological and Evolutionary Synthesis, University of Oslo, 0316 Oslo, Norway

## Abstract

The interconnection of the endocytic and autophagosomal trafficking routes has been recognized more than two decades ago with both pathways using a set of identical effector proteins and sharing the same ultimate lysosomal destination. More recent data sheds light onto how other pathways are intertwined into this network, and how degradation via the endosomal/autophagosomal system may affect signaling pathways in multicellular organisms. Here, we briefly review the common features of autophagy and endocytosis and discuss how other players enter this mix with particular respect to the Notch signaling pathway.

## 1. Introduction 

In eukaryotes, degrading cytoplasmic components is vital to the cells, since this process removes potentially toxic organelle remainders and protein aggregates, protects organisms from invading pathogens, and provides cells with nutrients by recycling the degraded macromolecules in periods of scarce food or under stress conditions. Macroautophagy (referred to as autophagy hereafter; other types of autophagy are not discussed here) has been shown to be the major degradation pathway, where cytosolic material is engulfed by double membrane vesicles (autophagosomes) and subsequently degraded after fusion with lysosomes [1, 2]. Despite its role as a survival pathway, autophagy also acts as a death mechanism implicated in protecting against cancer and neurodegenerative diseases [1, 3].

**Figure 1:**
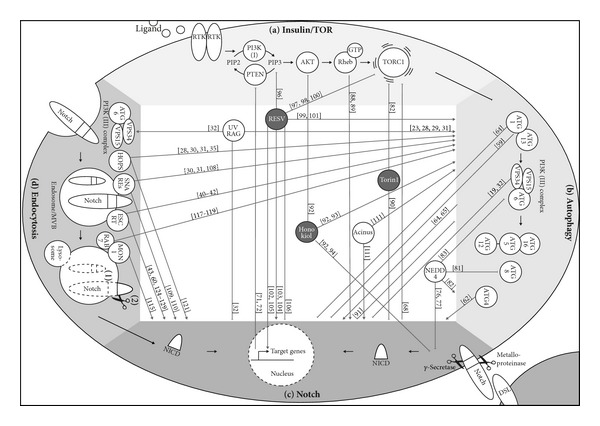
Network of the Insulin/TOR, autophagy, endocytosis, and Notch pathways. The Insulin/TOR pathway (a) normally inhibits the autophagic machinery (b). Notch signaling is induced by ligand (DSL) binding to the receptor and the cleaved NICD activates target gene expression (c). Alternatively, Notch can be activated ligand independently through receptor endocytosis ((d)2)), or the endocytosed receptor can be degraded to silence the signal ((d)1)). Connections of the pathways are shown between molecules directly or between molecules and the pathway in general (shaded areas). Bar-headed arrows indicate inhibition, arrows indicate activation. For simplicity, the trafficking routes are reduced to the processes and players discussed in the text. Numbers [] refer to publications cited in the text. Dark filled circles indicate drugs, whereas proteins are represented with white circles. RTK: receptor tyrosine kinase, RESV: resveratrol.

Autophagy can be activated by a wide range of signals but most intersect at the central regulator of autophagy and the target of rapamycin (TOR) complex 1 (TORC1, see Figure 1(a)). TORC1 inhibits autophagy by phosphorylating the core autophagy proteins associated into the Atg1 (autophagy-related gene 1) complex. Upon autophagy induction, this inhibition is released, and the activation of the Atg1 complex is followed by the assembly of proteins and lipids at the sites of autophagosome formation. This vesicle nucleation requires the activation of a class III PI3K (phosphoinositide 3-kinase) complex containing Vps34 (vacuolar protein sorting 34), Vps15, and Atg6/Beclin. Once initiated, the expansion and closure of the autophagic vesicle is mediated by two ubiquitin-like systems, Atg5-Atg12 and Atg8 (see Figure 1(b)). After completion, the autophagosome can fuse with compartments of the endocytic pathway, such as early endosomes, multivesicular bodies (MVBs), or late endosomes prior to fusion with lysosomes. Eventually, the autophagosomal cargo gets degraded by the acidic hydrolases of the lysosome and essential molecules such as amino acids are recycled and reused for cellular processes (for review, see [4–7]).

The endocytic system functions to internalize nutrients, macromolecules and plasma membrane compartments into the cell from outside by membrane invaginations and the formation of vesicles. These vesicles fuse to form early endosomes, from which proteins can be either recycled back to the plasma membrane through so called recycling endosomes, transferred to the trans-Golgi-network, form late endosomes or accumulate to MVBs. Finally, MVBs fuse with lysosomes for digestion and degradation of their content to provide them for cellular use. Endocytic processes are regulated by various proteins, such as clathrins, SNAREs (soluble NSF attachment protein receptor), Rab GTPases, the ESCRT (endosomal sorting complex required for transport), and HOPS (homotypic fusion and vacuolar protein sorting) complexes (reviewed in [8]) (see Figure 1(d)). In this respect, endocytosis acts as a complementary road to autophagy to provide the cell with amino acids and macromolecules.

Besides their common role in cellular nutrient supply and their shared terminal end point, the interconnection of both pathways is also demonstrated by the fact that autophagosomes fuse with early or late endosomes [9]. This collaboration in targeting vesicles for degradation may also be relevant for the regulation of cell signaling pathways. For example, epidermal growth factor receptor (EGFR) and Notch signaling were shown to require endosomal trafficking for activation, regulation, and degradation of the signal [10, 11] (Figure 1(c)). The evolutionary conserved Notch signaling pathway is fundamental in a wide range of cell types during several developmental and physiological processes, for example, by determining cell fate decisions required for neurogenesis and organogenesis or to control cell proliferation and cell death during development (reviewed in [12]). Given its pleiotropic function it is not surprising that dysregulation of Notch signaling leads to various diseases and forms of cancer (for a recent review see [13]). Thus, understanding the mechanisms regulating Notch signaling and the interconnection with other signaling pathways will be crucial to develop relevant therapeutic interventions.

## 2. Interconnection of Autophagy and Endocytosis

It has been postulated already in 1992 that endocytosis might be coupled to autophagy [14], and several studies have identified molecules that are used by both pathways for cellular degradation (for review, see [15]). We will shortly summarize the main common players present in both pathways and provide examples where autophagy and endocytosis are affecting each other.

### 2.1. Autophagy and Endocytosis Share the Same Effector Molecules

Given the fact that both autophagic and endocytic pathways are implicated in degrading cellular material, one would predict that autophagy and endocytosis partly use the same machinery. In fact, there is a striking conservation of regulatory factors between the two pathways, and many excellent reviews covering this aspect have been published (examples include [4, 15–18]). Here, we will focus on a subset of players with respect to recent publications demonstrating a link between endocytosis, autophagy, and the Notch signaling pathway (see below).

The nucleation of autophagosomes requires the activity of a class III PI3K complex, consisting of Vps34 (PI3K), Vps15, and Atg6/Beclin in yeast and higher eukaryotes [19, 20]. In mammals, the complex can be associated with other regulatory proteins, such as Atg14L/Barkor [21, 22], Rubicon (RUN domain and cysteine rich domain containing, Beclin1-interacting protein) [22], UVRAG (UV irradiation resistance-associated gene) [23], and Ambra1 (activating molecule in Beclin1-regulated autophagy) [24] to control the different functions of this complex. While the Ambra1-containing complex is required for the induction of autophagy [24], Atg14L/Barkor is thought to function in recruiting the Vps34-Vps15-Atg6 complex to the autophagosomal membrane to initiate autophagosome formation [25]. Rubicon, in contrast, negatively regulates autophagy and endocytosis by preventing the activation of Rab7, a protein that functions in lysosomal fusion and autophagosome maturation [22, 26, 27]. The role of UVRAG in autophagy remains controversial. Although it has been reported to positively regulate autophagy through overexpression in a cell line with monoallelic UVRAG deletion [23, 28], others could not detect a role for UVRAG in autophagy [21]. However, recent data supports a positive role of UVRAG by binding to BIF1 (also known as endophilin1), which was recently identified as a factor necessary for autophagosome formation [29]. UVRAG-associated complexes may promote autophagosome/endosome maturation, thus serving as a convergent point of both pathways [28]. Recently, it was shown that UVRAG influences later steps of autophagy by regulating the degradation of sequestered cargo in autolysosomes [30, 31]. Although orthologs of Atg14L and Rubicon exist in* Drosophila,* their function in the class III PI3K complex is not yet completely solved; however, recent data assigns dUVRAG a role in endocytosis [30–32].

In fact, the core class III PI3K complex containing Vps34, Vps15 and Atg6/Beclin is also found on early endosomes and is required for endocytosis (reviewed in [4, 8]). UVRAG, the accessory protein suspected to have a role in autophagosome maturation, has its primary role in the class III PI3K complex on endosomes, where it is necessary to activate the HOPS complex that mediates membrane tethering to fuse with other endosomes or lysosomes [21, 28]. UVRAG is normally bound and thereby inhibited by Rubicon. Upon activation, the small GTPase Rab7 competes for binding to Rubicon, which causes the release of UVRAG and its association with the HOPS complex involved in membrane tethering. The HOPS complex was shown to regulate tethering and fusion of endosomes to the vacuole in yeast as well as lysosomal delivery in* Drosophila* [33, 34]. Interestingly, the HOPS complex protein Dor was shown to control autophagosome maturation in* Drosophila* as well [35] (Figure 1). Thus, Rubicon and UVRAG may function both on endosomes and autophagosomes, while Atg14L and Ambra1 seem to be associated exclusively with the class III PI3K complex on autophagosomes.

However, a recent publication also links Atg14L to endocytic trafficking by demonstrating its binding to Snapin, a SNARE effector protein [36]. SNAREs are primarily involved in vesicle fusion events by assembling into trans-SNARE complexes with one v- (vesicle-) SNARE and three t- (target-) SNAREs on the donor and acceptor membrane, respectively. Several fusion events occur at different stages of autophagy to form autophagic structures, during maturation of autophagosomes and finally when autophagosomes unite with lysosomes. All these processes have been shown to involve the v- and t-SNARE proteins (for review, see [37]). Recently, autophagy was also assigned a role in secretion [38, 39], where SNAREs may mediate autophagosomal fusion with the plasma membrane in a similar way.

Finally, there is increasing evidence that another endosomal sorting complex is implicated in autophagy. ESCRT proteins were initially identified as major players in endosomal sorting and the formation of MVBs. Disruption of any of the 4 ESCRT complexes (0 to III) affects the fusion of late endosomes with autophagosomes, which has been studied extensively in models of neurodegenerative disorders (reviewed in [18]). Loss of function of the ESCRT machinery in* Drosophila* and mammals results in autophagosome accumulation and neurodegenerative diseases, most likely due to inhibited fusion with the endolysosomal system [40–42]. Interestingly,* Drosophila ESCRT* mutant cells show increased activation of JNK (c-Jun N-terminal kinase), a potent inducer of autophagy [43, 44]. Moreover, accumulation of autophagosomes and autolysosomes is also seen after knockdown of* ESCRT* components in mammalian cells [40, 41] (Figure 1). These findings in flies and mammals suggest that disrupting ESCRT function does not only inhibit autophagosomal-lysosomal fusion but may also induce autophagy.

ESCRTs have also been implicated in regulating other cellular functions, such as receptor signaling, cytokinesis, polarity, or migration. The sorting and degradation of components of various evolutionary conserved signaling pathways, such as EGFR, Hedgehog, or Wnt receptors, as well as Notch and its ligand Delta were shown to depend on the ESCRT machinery (reviewed in [45]).

### 2.2. Endocytosis Affects Autophagy and Vice Versa

Since the degradation of autophagosomal contents depends on lysosomes, the existence of a functional endocytotic pathway is fundamental to ensure the delivery of lysosomal hydrolases and the machinery to acidify vesicles to the lysosomal compartment. After digestion, the degradation products need to be able to exit the lysosome via specific transporters and permeases that are also provided by endocytosis [46]. Thus, endocytosis is a prerequisite for efficient autophagic flux.

On the other hand, there is increasing evidence that autophagy genes are required to ensure correct endocytic trafficking.* Drosophila Vps34* or* Atg6* (*Beclin*) mutant cells display defects in PI3P formation and fail to endocytose a fluid phase marker [19, 39]. This is in accordance with data from other systems where* Beclin* mutants were shown to be defective in endocytosis [47, 48]. Moreover, the UVRAG-containing Atg6 (Beclin) complex has recently been implicated in endocytic degradation in mammalian cells [49]. Interestingly, fluid phase endocytosis occurs normally in* Drosophila* cells mutant for* Atg1*, the core component necessary for autophagy induction, indicating that the lack of endocytosis in* Atg6* mutant cells is not due to defects in autophagy* per se* [39]. Remarkably, deletion of* Atg1*,* Atg6,* and* Vps34* also disrupts protein secretion, illustrating another example how autophagy participates in membrane trafficking events [39].

Several recent studies highlight the interplay of autophagy and endocytosis in the degradation of internalized gap junctions (for review, see [50]). Gap junctions (GJ) plaques are removed from the plasma membrane by endocytosis to reduce cellular adhesion in situations when cells need to detach from neighboring cells. Remarkably, vesicles containing endocytosed GJ are subsequently degraded by autophagy [51–54]. This degradation pathway is induced under several physiological and pathological conditions, such as during starvation in mouse and human cell cultures [51, 54], in failing canine cardiac tissue [53] or to remove accumulations of defective gap junction proteins to prevent cataract formation [54]. These studies also show that the ubiquitin-binding autophagy adapter protein p62 targets internalized GJs for degradation via the autophagosomal route [51, 54]. Thus, autophagy is necessary to complete the endocytosis of internalized gap junctions.

Lastly, another interesting connection between autophagy and endocytosis concerns Atg14L/Barkor, a positive regulator of autophagy. Atg14L/Barkor was shown to be able to act as a switch to guide the class III PI3K complex from its endosomal localization to the sites of autophagosome formation [25]. Taken together, these findings indicate a tight interconnection of the endosomal and autophagosomal trafficking routes.

## 3. When Endocytosis and Autophagy Converge at Notch

Endosomal trafficking acts as an organizer and modulator of many signaling events between and within cells at multiple stages of animal development (for comprehensive reviews, see [55, 56]). As mentioned above, endocytotic events are necessary to ensure proper receptor signaling in various conserved cellular pathways. This was extensively studied for the Delta-Notch pathway, which is activated by the binding of a DSL ligand (Delta, Serrate, or Lag-2) from the signal-sending cell to the Notch receptor on the signal-receiving cell. In brief, this binding triggers two proteolytic cleavages, the first mediated by a metalloprotease in the Notch extracellular domain (NECD) and a second by a *γ*-secretase complex within the transmembrane domain, which are important for internalization of the intracellular domain of the Notch receptor (NICD) and subsequent translocation to the nucleus where the NICD activates the transcription of target genes important for several developmental steps and cell fate decisions (for a comprehensive review, see [12]; for a glance, see [57] and see Figure 1(c)). To enable this activation process as well as for silencing of the signal, endocytosis plays a pivotal role, and mutations in vesicular trafficking components, such as dynamin disrupt the Notch signaling pathway [58]. Several steps during the maturation, signaling, recycling, and degradation process of both the ligand and the receptor require vesicle trafficking and endocytosis (reviewed in [11]) (see Figure 1(d)). As autophagic and endocytic pathways are tightly connected and share many effector proteins (see Section 2), autophagy might also be an important player in Notch signaling regulation.

We have previously reported a hitherto unexpected function of autophagy in receptor activation of the Notch signaling pathway during egg development in* Drosophila*, where the loss of autophagy leads to a precocious activation of the Notch pathway in the ovarian follicle cells [59]. In fact, the retention of Notch in endosomal vesicles accelerates its intramembranous cleavage and intensifies Notch signaling [60]. Given the interplay of endocytosis and autophagy, we propose that the absence of autophagy might lead to a pause in the normally rapid endosomal processing of internalized Notch, which in turn leads to pronounced NICD cleavage and enhanced Notch activity [59].

Led by these findings, we became increasingly aware of other observations within the literature that indicate different possible intersections between the autophagic and the Notch pathway—some, but not all also related to endocytosis (Figure 1). Three possible mechanisms of interaction between both pathways could be found. In the first scenario, autophagy influences the Notch signaling pathway. Secondly, the Notch pathway affects autophagy and, finally, a “factor-X” impacts on both autophagy and Notch.

In the following sections we will give a short overview of these findings without discussing them in details, as the diversity of topics would go beyond the scope of this review. Instead, we hope to provide new aspects for further research regarding the interplay of endocytic trafficking, the autophagic, and the Notch signaling pathways.

### 3.1. Influence of Autophagy on the Notch Signaling Pathway

The Notch pathway is important during animal development, and defective signaling can result in malformed tissues such as the “notched-wing” phenotype in* Drosophila*, originating by impaired Notch signaling along the dorsoventral wing disc boundary [61]. The first direct observation of an* Atg* mutation impairing Notch signaling was made by Thumm and Kadowaki [62], who described that deletion of the cysteine protease Atg4 in* Drosophila* enhances the notched-wing phenotype induced by loss of function of components of the Notch pathway. However, the mechanism by which the lack of autophagy might influence Notch signaling remained unclear. In a more recent study the* Drosophila* ATPase Domino (Dom), a positive regulator of Notch signaling [63], was used to screen for modifiers of the Dom induced notched-wing phenotype [64]. Kwon and coworkers discovered that loss of* Atg1* restores normal development of the wing whereas overexpression of Atg1 enhances the notched-wing phenotype [64]. Surprisingly, the downregulation of other* Atg* genes showed the opposite result and enhanced the notched-wing phenotype similarly to the results obtained for* Atg4* mutants [62], indicating a secondary function for Atg1 in addition to its general function in autophagy [64].

Dysregulated Notch signaling can also lead to wing-vein patterning defects [61], and downregulation of autophagy by Atg-RNAi in the* Drosophila* wing was shown to result in loss of vein tissue and/or ectopic vein patches [65]. In contrast, our results showed that expression of Atg1- and Atg5-RNAi in the wing did not induce any vein defects but led to slightly larger posterior wing compartments (unpublished data). However, these varying results might be due to different expression techniques and RNAi lines used, leading to variable levels of autophagic downregulation.

Notch signaling is also required during* Drosophila* oogenesis and egg chambers containing* Notch* mutant follicle cells display a wide range of developmental defects [66]. Similar defects were also found in egg chambers lacking autophagy, and mutations in* Atg1* or* Atg13* were found to induce precocious activation of the Notch pathway as shown by the expression of the Notch downstream effectors Cut and Hindsight (Hnt) [59].

Taken together, these results hint at a function for autophagy within the Notch pathway; however the underlying mechanisms are still obscure and unexplored. The fact that loss of autophagy seems to intervene in a positive as well as negative way with Notch activity renders a definite conclusion difficult. The variable effects seen might be due to secondary functions of specific* Atg* genes, specialized functions in different organs, tissues, or developmental stages. For example, Notch signaling in the* Drosophila* ovaries is activated by Delta expression in the germline within a defined time frame. Autophagy deletion affected Notch activity only during that short period of activation, most likely by regulating the processing of the endocytosed Notch receptor, which presumably can be compensated for by other degradation pathways in later stages of development [59].

### 3.2. Influence of the Notch Pathway on Autophagy

Interestingly, Notch signaling was also discovered to be important for regulating the autophagic pathway. A recent study resulted from work on the nematode* C. elegans*, a species with two* Notch* genes:* lin-12* and* glp-1* [67]. Mutants of* glp-1* were shown to exhibit decreased fat storage, defective germline proliferation, and increased autophagy levels [68–70]. Lapierre and coworkers suggest that autophagy in these mutants is induced at a transcriptional level, possibly via downregulation of TOR (Figure 1) [68]; however, so far no further investigations to confirm a link between the loss of Notch signaling activity and the upregulation of autophagy in* glp-1* mutants have been carried out.

In another study, Palomero et al. [71] investigated activating* Notch* mutations in T-cell leukemia (T-ALL) and were able to show that Notch regulates the expression of PTEN (phosphatase and tensin homolog; see Figure 1(a)) through activation of the Notch target gene* Hes1* (hairy and enhancer of split-1), which induces the activity of the class I PI3K-AKT signaling pathway in leukemic T cells, but also in normal thymocytes. Inhibition of Notch signaling within this system did indeed induce autophagy. PTEN downregulation by Notch-induced expression of Hes1 was also shown in DN3 thymocytes [72]; however, autophagy was not examined in this system. This regulatory link seems to be functionally conserved since class I PI3K-AKT activation is also necessary for Notch induced growth in* Drosophila* [71]. Despite the control via Hes1, other mechanisms for a regulation of class I PI3K through Notch seem to exist (for a review see [73]), but regulation of autophagy via the Notch—class I PI3K pathway has not been directly tested so far.

### 3.3. “Factor X” Influences Both Autophagy and Notch

Several studies identified factors that are intertwined between both pathways and either concomitantly regulate Notch and autophagy signaling or regulate one through the other by a yet unknown mechanism. One example is the E3 ubiquitin ligase Nedd4 (neural precursor cell-expressed developmentally downregulated 4) family known to regulate trafficking and endosomal degradation of multiple target substrates within different cellular environments (reviewed in [74]). It has been shown that both* Drosophila* Nedd4 ligases “suppressor of deltex” (SU (DX)) [75] and Nedd4 are capable of negatively regulating Notch mediated signaling by direct ubiquitination and subsequent degradation of Notch [76, 77]. Another E3 ubiquitin ligase, Mib1 (mindbomb homolog 1), acts on the Notch ligands Delta and Jagged to promote their endocytosis, leading to reduced Notch1 activity in mammals [78–80]. Recently, a proteomic analysis of the autophagy interaction network in human cells revealed that Nedd4 also associates with multiple Atg8 (LC3) proteins, possibly to target Atg8 for degradation [81]. In line with this, the interaction between Nedd4 and Atg8 orthologs was strongly reduced after treatment with Torin1, a TOR inhibitor [82], indicating that degradation of Atg8 by Nedd4 is decreased upon autophagy induction by inhibition of TOR. In addition, Nedd4 depletion led to an increase in autophagosome formation [81]. Furthermore, Nedd4 was also found to negatively control the stability of Beclin1, a protein important for the initiation of autophagy [83] (Figure 1). As discussed above and reviewed by Falk and coworkers [50], autophagy and endocytosis are concomitantly implicated in the degradation of internalized gap junctions. Notably, Nedd4 mediated ubiquitinylation seems to be required for the targeting of the autophagic machinery and subsequent autophagic degradation of gap junction connexins [51]. It therefore appears that shared ubiquitination proteins regulate both autophagy and Notch signaling. In this respect, it was shown that Wnt signaling is regulated by autophagy through specific degradation of its ligand dishevelled (Dvl). Ubiquitination of Dvl facilitates its binding to the autophagic receptor p62 which allows LC3-mediated autophagic degradation of Dvl to downregulate Wnt signaling [84]. A similar scenario could be envisioned for Notch and its ligand Delta, in which Delta is selectively ubiquitinated (e.g., by Mib1) and subsequently degraded by autophagy to downregulate Notch signaling.

The canonical Insulin/TOR pathway is well established as a regulator of growth and autophagic activity in various systems [85] and activation of this pathway by, for example, overexpression of the GTP-binding protein Rheb (Ras homolog enriched in brain) has been shown to activate growth and inhibit autophagy in multiple tissues in* Drosophila* [86, 87]. Surprisingly, two recent publications have indicated that the Insulin/TOR pathway might also be responsible for alterations in Notch signaling [88, 89]. Karbowniczek and coworkers found that Rheb overexpression in the asymmetrically dividing* Drosophila* external sensory organ produces a cell fate switch from neuronal development to the development of only hair and socket cells, a phenotype consistent with ectopic Notch activation [88]. Ma and coworkers [89] used mouse and human cells to also show that activated Rheb induced enhanced Notch signaling (Figure 1). TOR inhibitors were able to block Notch activation in these cells; however, this was not observed in* Drosophila* [88], hinting to a cell-type specific mechanism where Notch activation by Rheb is TOR independent. In addition, expression of Notch, but not the Notch target gene* Hes1*, was significantly reduced in mice tumor tissue treated with the TOR inhibitor Torin1 [90]. Furthermore, when the team of Ge and Ren [91] investigated the effects of alcohol intake on myocardial damage in aldehyde dehydrogenase-2 (Aldh2) overexpressing transgenic mice, they discovered that alcohol intake triggered myocardial autophagy and inhibited Notch signaling in wild type but not transgenic mice. The observation that Aldh2 seems to protect against alcohol induced inhibition of AKT, TOR, and STAT3 (signal transducer and activator of transcription 3) phosphorylation and the fact that additional inhibition of Notch signaling intensified autophagy lead the authors to the speculation that mTOR-STAT3 signaling and subsequent Notch activation inhibited autophagy induction [91] (Figure 1). These results show that autophagy and Notch signaling might—in some situations—both respond to changes in the activity of the Insulin/TOR pathway.

More evidence for a common regulatory mechanism for Notch and autophagic signaling emerges from a number of anticancer drug studies. The drug Honokiol, which was shown to increase autophagosome formation [92] and the expression of autophagic markers like Beclin1 and lipidated Atg8 [93], was also found to reduce Notch signaling, possibly by inhibition of the *γ*-secretase complex [92, 94]. Furthermore, Honokiol was also potent to inhibit AKT and TOR [92], suggesting again a role of Insulin/TOR signaling for the regulation of autophagy and Notch (Figure 1).

Another compound claimed to have protective effects on various diseases is resveratrol (for review, see [95]). It has been shown to negatively regulate TOR signaling by directly inhibiting class I PI3K [96] or AMPK (AMP-activated protein kinase) [97] but also TOR through the increase of its association to the inhibiting protein DEPTOR (DEP domain-containing mTOR-interacting protein) [98]. Resveratrol induced activation of autophagy via AMPK seems to depend on the cell type, as in human esophageal squamous cell carcinoma autophagy induction is independent of AMPK/TOR signaling [99] whereas in human lung carcinoma cells it is not [100]. However, the general potency of resveratrol to activate autophagy is strongly supported by various mechanisms, including Beclin-independent induction, regulation of AKT, mTOR, and AMPK activity, or though the activation of JNK or p62 pathways (for reviews, see [100, 101]). In addition, resveratrol was found in a high-throughput screen for the identification of Notch activating compounds using carcinoid cell lines [102], and treatment of thyroid carcinoma cells with resveratrol was shown to inhibit growth by arresting cell-cycle progression in the S-phase and induce Notch protein expression and signaling by transcriptional regulation [103, 104]. Furthermore, resveratrol also suppressed cellular growth and activated Notch expression in glioblastoma [105]. In contrast to most other studies, a microarray analysis to search for genes expressed in adipose tissue of obese men treated with resveratrol found genes of the Notch signaling pathway to be downregulated, whereas autophagy genes were found to be upregulated [106]. However, most studies agree on an activating effect of resveratrol on autophagy as well as on Notch signaling (Figure 1), which is probably interconnected with its growth inhibiting function that may as well depend on the cellular context.

As described above, the functionality of both autophagy and Notch signaling requires endocytic vesicle trafficking and fusion, the latter of which is often mediated by SNAREs (for a review, see [107]). SNAREs play a role in nearly all steps of autophagy, from the formation of the autophagosome, to its maturation and the autophagosome-lysosome fusion (reviewed in [37]). For example, loss of the SNARE protein Syntaxin 17 in* Drosophila* is followed by an accumulation of mature autophagosomes, indicating the requirement of Syntaxin 17 for the fusion with lysosomes [31, 108]. Interestingly, the interaction of Syntaxin 17 with the HOPS complex seems to be required for fusion of autophagosomes with lysosomes both in* Drosophila* and mammals [30, 31]. Evidence for a role of SNARE protein family members in Notch signaling was found in* Drosophila* by expressing mutant forms of Syntaxin in the developing wing, resulting in Notched wings indicative for a disruption of the Notch pathway [109]. In addition, trafficking defects were observed in mutants for Avalanche, another Syntaxin-family protein, leading to increased abundance of the Notch receptor at the cell surface and at peripheral structures [110] (Figure 1).

Another link between autophagy, endocytosis, and Notch is the* Drosophila* protein Acinus (dacn), which plays a role in endosomal trafficking of signaling receptors such as Notch and the EGF receptor. Loss of dacn inhibits autophagosome maturation into autolysosomes, thus blocking the autophagic pathway, but also destabilizes early endosomes to enhance the delivery of endocytosed receptors to lysosomes, which leads to an inhibition of Notch and EGFR signaling [111]. Thus, dacn might regulate the fusion of autophagosomes with endosomes, a step that is crucial for the maturation of autophagosomes, assigning dacn yet another role in the complex interplay between endocytosis and autophagy. The maturation of autophagosomes into autophagolysosomes presents another step in which endosomal and autophagosomal pathways may converge to modulate Notch signaling. Reagents that elevate lysosomal pH and thus block lysosomal function (e.g., chloroquine or bafilomycin A) also inhibit autophagic degradation and are commonly used to determine autophagic activity [112]. Interestingly, the vacuolar proton pump responsible for acidification of intracellular compartments (V-ATPase) was shown to be required for Notch signaling and endocytic trafficking in* Drosophila.* Acidification of endocytic vesicles by V-ATPase enhances Notch degradation and signaling in endosomes, suggesting that the acidic endosomal environment is a prerequisite for efficient Notch activation in the signal-receiving cell [113, 114].

In Section 2.1, we describe the importance of the class III PI3K complex containing Vps34, Vps15, and Atg6/Beclin, and—depending on the function—also UVRAG or Atg14L and Ambra1, and how its members are involved in autophagy and endocytosis. Notably,* UVRAG* and* Vps34* mutant flies are both defective in autophagy and accumulate endosomal Notch [19, 32], which can lead to enhanced Notch activity [32]. However, the requirement of UVRAG in autophagy is most likely restricted to later steps of endocytic degradation, because autophagosomal-lysosomal fusion occurs normally in* UVRAG* mutant flies and* UVRAG* depleted mammalian cells [30, 31]. An accumulation of Notch in large endosomes could also be shown in flies mutant for Dmon1 (the* Drosophila* ortholog of Mon1/Sand1; [115], Figure 1), a protein essential for the exchange of Rab5 with Rab7 on maturing endosomes [116]. Rab7 and yeast Mon1 were both also found to be important for autophagy [117–119]; however, regulation of autophagosomes seems not to be affected in* Dmon1* mutant flies, nor was enhanced Notch signaling observed [115].

Rab7 also interacts with the heterohexamer membrane tethering complex HOPS to promote membrane fusion (for reviews, see [107, 120]) and HOPS is required for fusion of autophagosomes with the lysosome in* Drosophila* [35], and together with UVRAG for autophagosome maturation and endosomal fusion in humans [28]. Furthermore,* Drosophila* HOPS contributes to Notch signaling by delivering endocytosed Notch to the lysosome where internal parts are degraded, but extracellular parts can contribute to signaling [121] (Figure 1). Two recent papers demonstrate the requirement for HOPS in autophagosome-lysosome fusion [30, 31], and mutants of several subunits of the* Drosophila* HOPS complex accumulate Notch in the developing eye [31]. In the model proposed by Wilkin and coworkers [121], the NECD of the Notch receptor at the limiting membrane of late endosomes is removed by internal lysosomal proteases, whereas the NICD is released. This mechanism could also explain enhanced Notch activity seen in mutants defective for autophagy or endosomal trafficking [32, 59] where Notch could get similarly lodged due to discontinued degradation pathways.

Finally, the ESCRT complex, acting as cargo sequestering and sorting machinery for multivesicular bodies (MVBs), has well-established functions in autophagy (see Section 2 and for a review [18]), but also in the receptor downregulation of several cellular signaling events (reviewed in [122]). For silencing, transmembrane receptors like Notch are cleared from the membrane and delivered to endosomes, where they become ubiquitinated, and invaginated as MVBs before fusion with the lysosome and degradation. In ESCRT mutants, biogenesis of MVB cannot take place and receptors accumulate on enlarged endosomes (for a review on Notch activation, see [123]). Accumulation of the Notch receptor in endosomal compartments and ectopic signaling activity has been shown in cells mutant for components of the ESCRT-I, -II, or -III complex [43, 60, 124–129]. Increased Notch signaling activity in ESCRT mutant cells is presumably caused by the prolonged duration for which Notch and its ligand are trapped in endosomal compartments and are accessible for *γ*-secretase cleavage [43, 60, 126–128] (Figure 1); however, ligand independent activation of the pathway as described above [121] or ligand activation dependent of the ubiquitinylation status of Notch is also possible [125].

## 4. Summary

Several proteins are implicated in both autophagy and endosomal receptor sorting, and numerous intersections between the endosomal and autophagic pathways have been described. Despite the established role of endocytosis in sorting, recycling, and degradation of signaling receptors and their ligands, there is increasing evidence that autophagy is also involved in executing these events. Cells may regulate the trafficking of activated receptors to ensure adequate levels of signaling and to modulate the signal strength.

The activity of many pathways relies on correct processing of their receptors and/or ligands, as shown for EGFR and Notch, two evolutionary conserved key signaling pathways implicated in the development of higher eukaryotes. Given the fact that the dysregulation of Notch signaling contributes to tumor growth (reviewed in [13]), the described interconnections to the autophagy pathway will be of significant interest to exploit autophagic processes for cancer therapy.

By summarizing recent findings on the emerging network between autophagy, endocytosis, and the regulation of Notch signaling, we hope to establish novel starting points for further research in this area.

## List of Abbreviations

Aldh2: Aldehyde dehydrogenase-2
Ambra1: Activating molecule in Beclin1-regulated autophagy
AMPK: AMP-activated protein kinase
ATG: Autophagy related genes
DEPTOR: DEP domain-containing mTOR-interacting protein
DSL: Delta, serrate, or lag-2
Dvl: Dishevelled
EGFR: Epidermal growth factor receptor
ESCRT: Endosomal sorting complex required for transport
GJ: Gap junctions
Hes1: Hairy and enhancer of split-1
Hnt: Hindsight
HOPS: Homotypic fusion and vacuolar protein sorting
JNK: c-Jun N-terminal kinase
Mib1: Mindbomb homolog 1
MVB: Multivesicular bodies
NECD: Notch extracellular domain
Nedd4: Neural precursor cell-expressed developmentally downregulated 4
NICD: Notch intracellular domain
PIP2: Phosphatidylinositol 4,5-bisphosphate
PIP3: Phosphatidylinositol (3,4,5)-trisphosphate
PI3K: Phosphoinositide 3-kinase
PTEN: Phosphatase and tensin homolog
RESV: Resveratrol
Rheb: Ras homolog enriched in brain
RTK: Receptor tyrosine kinase
SNAREs: Soluble NSF attachment protein receptor
STAT3: Signal transducer and activator of transcription 3
SU (DX): Suppressor of deltex
Rubicon: RUN domain and cysteine-rich domain containing Beclin1-interacting protein
TOR: Target of rapamycin
UVRAG: UV-resistance associated gene
V-ATPase: Vacuolar H^+^-ATPase
Vps: Vacuolar protein sorting.
